# Genetic variability of human metapneumo- and bocaviruses in children with respiratory tract infections

**DOI:** 10.1111/irv.12185

**Published:** 2013-11-07

**Authors:** Vasiliki Pogka, Afroditi Moutousi, Athanasios Kossyvakis, Antonios Kalliaropoulos, Dionyssios N Sgouras, Maria Giannaki, Andreas F Mentis

**Affiliations:** a National Influenza Reference Laboratory of Southern Greece, Hellenic Pasteur InstituteAthens, Greece; b Laboratory of Medical Microbiology, Hellenic Pasteur InstituteAthens, Greece; c Department of Microbiology, Aghia Sophia Children's HospitalAthens, Greece

**Keywords:** Bocavirus, children, metapneumovirus, phylogenetic analysis, respiratory viruses

## Abstract

**Objectives:**

The genotypic analysis of human metapneumo-(HMPV) and boca-(HBoV) viruses circulating in Greece and their comparison to reference and other clinical strains.

**Design:**

Genetic analysis of representative strains over three consecutive winter seasons of the years 2005–2008.

**Setting:**

Representative positive specimens for HMPV and HBoV from paediatric patients of healthcare units and hospitals in Southern Greece with influenza-like illness or other respiratory tract infections.

**Sample:**

Seven to ten positive specimens for either HMPV or HBoV from each winter period. In total, 24 specimens positive for HMPV and 26 for HBoV, respectively.

**Main outcome measures:**

Sequence diversity of HMPV and HBoV strains by sequencing the complete G and VP1/VP2 genes, respectively.

**Results:**

In total, 24 HMPV strains were found to have a 92–100% nucleotide and a 85.9–100% amino acid identity. Phylogenetic analysis based on the number of amino acid differences, revealed circulation of 4 different subclusters belonging to genetic lineage B2. Similarly, analysis of 26 HBoV strains indicated that 22 clustered within genotype St2, 2 into genotype St1 and the remaining 2 formed a third cluster derived from potential recombination between different St1 genotype strains. St2 HBoV genotype was observed throughout the whole observation period whereas St1 only during the second and the third winter period. Higher levels of heterogeneity were observed between HMPV compared to HBoV strains.

**Conclusions:**

Phylogenetic analysis revealed circulation of one single lineage (B2) for HMPV viruses and predominance of St2 genotype for HBoV viruses. A possible recombination between St1 genotype strains of HBoV was observed.

## Introduction

Newly discovered viruses, such as human metapneumovirus (HMPV) and human bocavirus (HBoV) are important pathogens causing respiratory tract infection (RTI) in susceptible populations, particularly in children and the elderly.^1^–^4^

HMPV is a negative single stranded RNA virus belonging to the family of *Paramyxoviridae,* which was first isolated in 2001^5^ from nasopharyngeal aspirates. It is responsible for about 5–15% of the worldwide respiratory tract infections, affecting both young children and adults, causing symptoms ranging from mild disease of the upper respiratory tract system to severe bronchiolitis and pneumonia.^2^ In infants, HMPV incidence is even higher, reaching 25%.^6^ Genetic analysis of HMPV isolates revealed two ‘major’ lineages (A and B) and four ‘minor’ lineages (A1, A2, B1 and B2), based on the sequence variability of the attachment (G) and fusion (F) surface glycoproteins. The existence of two further sublineages, A2a and A2b, has further been suggested.^7^

HBoV, a single stranded DNA virus belonging to the family of *Parvoviridae*, was discovered in 2005^8^ and has been detected in nasopharyngeal aspirates, sera and blood samples of patients with respiratory tract infection and in faecal specimens of patients with acute respiratory illness and/or gastroenteritis. HBoV affects, mostly, children younger than 2 years old, with its incidence ranging from 2·7 to 19% in respiratory and from 0·8 to 9·1% in faecal samples, obtained from patients with respiratory infection and gastroenteritis, respectively.^9^ Four species of HBoV have been recently discovered with HBoV1 being predominantly a respiratory pathogen, whereas HBoV2, HBoV3 and HBoV4 being found mainly in stool.^10^ Nucleotide sequence analysis of the two capsid proteins (VP1 and VP2), which show high variability compared with coding sequences of the two viral nonstructural proteins (NS1 and NP1), has further divided HBoV1 into genotypes St1 and St2. These two major HBoV genotypes correspond to the original St1 (Stockholm 1) and St2 (Stockholm 2) isolates.^8^

Epidemiological and phylogenetic analysis of HMPV and HBoV has been carried out extensively in other countries but not in Greece, where only a few studies concerning newly recognized respiratory viruses have been published. Using molecular methods for identification, we recently reported the incidence of 13 respiratory pathogens, including HMPV and HBoV, in children presenting with influenza-like illness (ILI).^11^ In this study, we aimed to determine the genetic diversity of the HMPV and HBoV clinical strains identified within the Greek population by comparison with reference, as well as, wild strains described in other countries.

## Patients and methods

### Clinical specimens

Clinical specimens including nasopharyngeal aspirates and/or throat swabs were collected over the winter seasons (November to May) of 2005/2006, 2006/2007 and 2007/2008 by paediatricians in healthcare units of Southern Greece and in collaborating paediatric hospitals of Athens. Specimens were sent to the National Influenza Reference Laboratory of Southern Greece in viral transport medium (Mediaproducts BV, Groningen, The Netherlands). Our sample group consisted in total of 3306 specimens from paediatric patients aged 0–18 years-old, 1272 out of whom suffered from ILI (sudden onset of symptoms, with high fever and cough in the absence of other diagnosis) and the remaining 2034 from other upper and lower respiratory tract infections which did not fall within the definition of ILI. The presence of HMPV RNA and HBoV DNA concerning patients with ILI has been described in our previous study.^11^ In the present study, the same PCR protocols were used to reveal the presence of HMPV and HBoV in patients with other upper and lower respiratory tract infections (data not shown). Moreover, all specimens found negative for HBoV1 in our previous study^11^ were further screened for the three recently identified species, HBoV2, HBoV3 and HBoV4, as previously described.^12^

### Nucleotide sequence analysis of HMPV and HBoV strains

In total, presence of HMPV RNA was detected in 188 (5·7%) and HBoV DNA was detected in 193 (5·8%) cases of the 3306 patients screened. Seven to ten positive specimens for either HMPV or HBoV belonging to each winter period (in total 50 samples) were further studied using specific primers mapping highly variable regions of the viral genome. The selected specimens amounting to 13% of total positive strains corresponded to the beginning (November-December), middle (January-February) and end (March-May) of each winter season. More specifically, 24 samples positive for HMPV were further amplified to obtain the genomic region corresponding to the 969 bp DNA fragment, encompassing the full-length of G protein coding region (240 amino acids).^13^ Likewise, 26 samples positive for HBoV were amplified utilizing 4 overlapping sets of primers, spanning the whole length (672 amino acids) of the coding region corresponding to the VP1 and VP2 proteins (Table 1).^14^

**Table 1:** Primers sequences for sequencing analysis used in this study

Primer	Sequence (5′-3′)	Target	Position (5′-3′)	Reference citation
HMPV F1	TACAAAACAAGAACATGGGACAAG	G	6183–6206*	13
HMPV R1	GAGATAGACATTAACAGTGGATT	G	7127–7149*	13
HBoV F1	GATAACTGACGAGGAAATGCT	VP1/VP2	3009–3029**	14
HBoV R1	AGTATGTCCATGGAGTTGTGA	VP1/VP2	3711–3731**	14
HBoV F2	TTCAGAATGGTCACCTCTACA	VP1/VP2	3639–3659**	14
HBoV R2	CTGTGCTTCCGTTTTGTCTTA	VP1/VP2	4266–4286**	14
HBoV F3	AACTTTGACTGTGAATGGGTTA	VP1/VP2	4172–4193**	14
HBoV R3	AAATAGTGCCTGGAGGATGAT	VP1/VP2	4767–4787**	14
HBoV F4	ACCAAGGGCTGACAAACACA	VP1/VP2	4711–4730**	14
HBoV R4	TGTACAACAACAACACATTAAAAG	VP1/VP2	5276–5299**	14

* Numbering according to reference FJ168778.

** Numbering according to reference DQ000496.

PCR products were purified using the QIAquick PCR purification kit (Qiagen, Hilden, Germany) and the MinEluteTM gel extraction kit (Qiagen) and subjected to direct sequencing in both directions utilizing the GenomeLab DTCS-quick start sequencing kit (Beckman Coulter, Brea, CA, USA) on a CEQTM 8000 genetic analyser (Beckman Coulter).

### Phylogenetic analysis

Sequences obtained were examined in terms of closest homology sequence using blast software (http://www.ncbi.nlm.nih.gov/BLAST/). Multiple sequence alignments of representative strains identified in Europe, in addition to GenBank sequence data from original HMPV and HBoV prototype strains were made by BioEdit sequence alignment editor. Phylogenetic analysis of HMPV and HBoV-positive samples was performed using the molecular evolutionary genetics analysis (MEGA) software, version 5.^15^ Phylogenetic trees were constructed by the neighbour-joining method (NJ, tree algorithm inferred with the Kimura 2-parameter substitution model of sequence evolution)^16^,^17^ while, a bootstrap resampling analysis was performed (1·000 replicates) to test tree robustness.^18^ Moreover, comparison of the mean sequence diversity in pairwise alignments was applied on both our strains and the representative prototypes. More specifically, the aligned sequences were compared using uncorrected p-distances (proportion of observed differences), handling gaps with complete deletion.

### Nucleotide sequences accession numbers

All 50 sequences determined in the present report have been deposited in the GenBank/EMBL/DDBJ database under accession numbers JQ513461–JQ513510. The reference strains used in this study have been previously assigned with the following accession numbers: a) for the Netherlands HMPV prototypes AY296040 (NL/94/01), AY296034 (NL/1/99), AF371337 (NL/1/00), AY296021 (NL/00/17) b) for Stockholm HBoV prototypes DQ000495 (St1), DQ000496 (St2).

## Results

### Phylogenetic analysis of HMPV sequences

**Figure 1:**
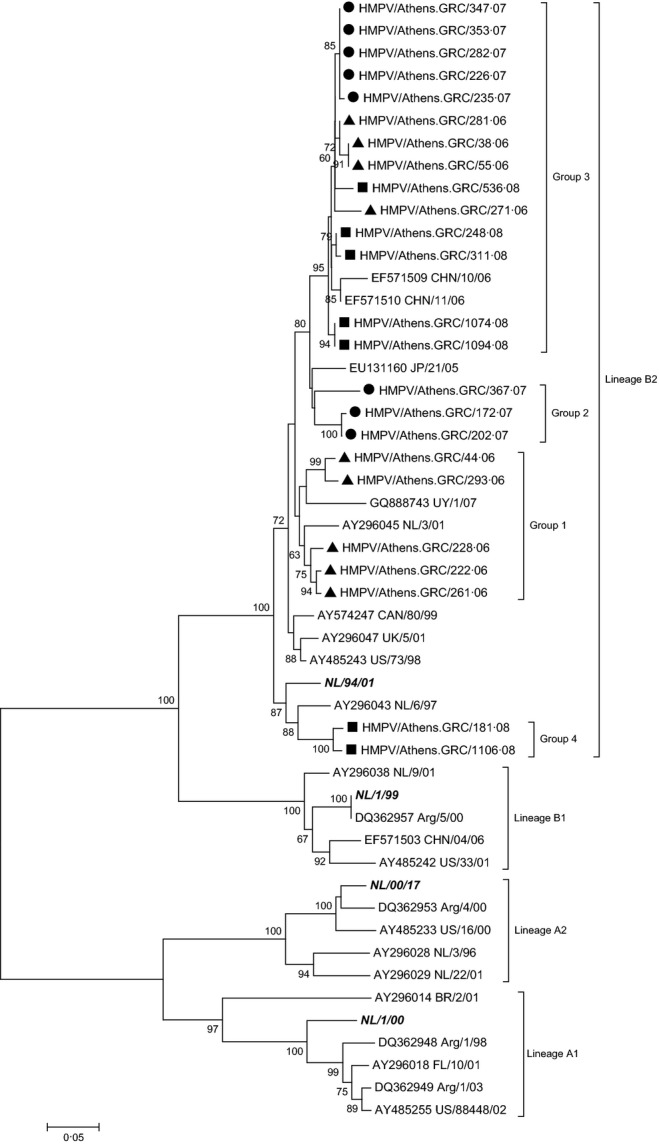
Evolutionary relationships of Greek HMPV strains with reference genotypes based on the complete coding sequence of G gene. The evolutionary history was inferred using the Neighbor-Joining method. The percentage of replicate trees in which the associated taxa clustered together in the bootstrap test (1000 replicates) is shown next to the branches. Only values above 60% are shown. Strains isolated in 2006 (▲), 2007 (•) and in 2008 (▪) are depicted. Reference strains are indicated in italics.

Twenty-four HMPV-positive samples, including 9 isolates detected in 2006, 8 detected in 2007 and 7 isolates detected in 2008, were characterized at the molecular level and a phylogenetic tree of the complete G gene was generated. Comparison with the prototype strains from the Netherlands^13^ revealed that all Greek-HMPV isolates clustered with NL/94/01 in genetic lineage B2 (Figure 1) and presented a nucleotide (nt) identity of 92·0–100·0% and an amino acid (aa) identity of 85·9–100·0% to each other.

**Figure 2:**
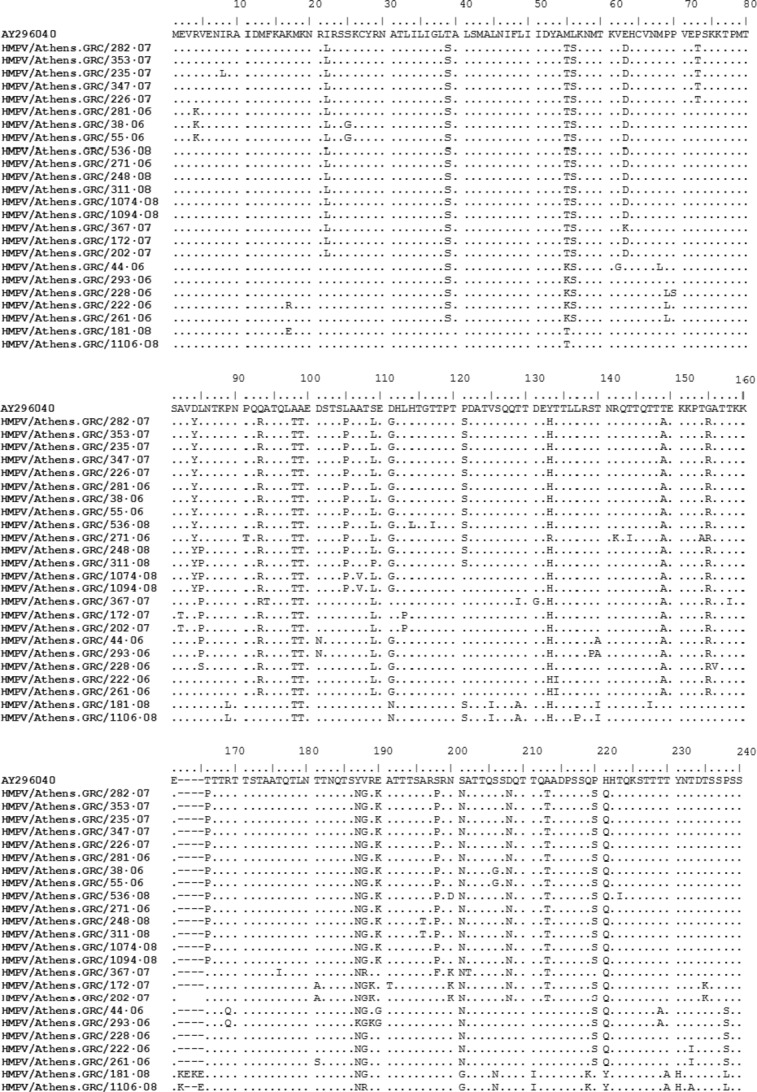
Deduced amino acid sequences of the G protein of 24 Greek HMPV strains. Prototype strain AY296040 is displayed as reference sequence for lineage B2. Dots indicate identical amino acid and dashes correspond to gaps.

Phylogenetic analysis of the studied strains classified them within 4 separate groups based on the number of aa differences they shared. More specifically, group 1 included HMPV strains that circulated during the first winter period and shared an aa substitution at position 238 (P238S), group 2 included strains which circulated during the second winter season and shared an aa substitution at position 200 (N200K) and group 3 included strains that circulated during the whole period under investigation and revealed 5 aa substitutions (D84Y, L105P, T166P, E190K and S198P). Concerning group 4, sequence alignment revealed the insertion of 2 additional amino acids in specimen 1106/08 (one Lys and one Glu) and 4 amino acids in specimen 181/07 (two Lys and two Glu). These two HMPV strains also shared a number of amino acid substitutions in relation to all the other Greek specimens analysed in this study (Figure 2).

Intergroup analysis exposed 4 aa substitutions between groups 2 and 3 (I22L, E63D, D208N and A213T), 7 aa substitutions between groups 1, 2 and 3 (T39S, L56S, Q93R, S109L, T149A, G155R and P220S) and 1 aa substitution between groups 1, 3 and 4 (D111G/N). Comparing the Greek strains with the HMPV B2 reference sequence, they all shared 8 aa substitutions throughout the whole G protein (M55T/K, A98T, A99T, Y133H/R, Y187N/K, V188G/R, S201N/G, H221Q/Y).

To summarize, sequencing analysis suggested that among HMPV strains, only those belonging to group 3 circulated throughout the whole period under study. Intragroup analysis within group 3 also suggested specific variations within the strains that circulated during each winter season. More specifically, all 2007 strains revealed a proline to threonine aa substitution at position 73 (P73T) and a proline to serine aa substitution at position 121 (P121S). The P121S substitution was also observed in all strains from 2006 and in 3 strains from 2008. Within each winter period, no sequence variations were observed among strains in each group.

### Phylogenetic analysis of HBoV sequences

**Figure 3:**
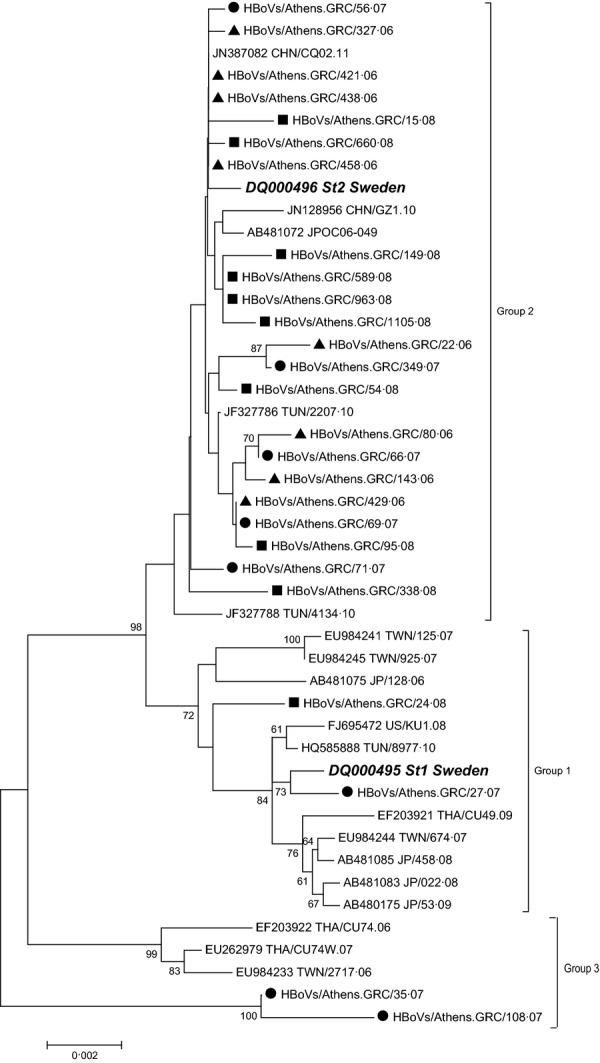
Evolutionary relationships of the Greek HBoV strains with reference genotypes using the complete coding sequence of VP1/VP2 gene. Evolutionary history was inferred using the Neighbour-Joining method. The percentage of replicate trees in which the associated taxa clustered together in the bootstrap test (1000 replicates) is shown next to the branches. Only values above 60% are shown. Strains isolated in 2006 (▲), 2007 (•) and in 2008 (▪) are depicted. Reference strains are indicated in italics.

Twenty-six representative HBoV-positive samples were analysed using the complete VP1/VP2-gene region. Eight of these samples were detected in 2006, 8 in 2007 and the remaining 10 samples in 2008. Phylogenetic analysis of the Greek-HBoV strains indicated that 2 (7·7%) of them clustered into genotype St1 and 22 (84·6%) into genotype St2 (Figure 3). The remaining 2 (7·7%) strains studied, HBoVs/Athens.GRC/35.07 and HBoVs/Athens.GRC/108.07, which clustered neither with St1 nor with St2 genotype, formed a third group which shared 98·2–98·5% nt identity with St1 and 98·4–98·7% nt identity with St2. All of the Greek strains showed an nt identity of 98·2–100·0% and a deduced aa identity of 95·6–100·0% with each other and appeared to be most closely related to the Tunisian (TUN2207) and the Chinese (CQ201102) strains.

More specifically, for specimens belonging to St1 and St2 groups, analysis of intra- and intergenotype nt and aa sequences revealed different levels of homology. When comparing strains belonging to the above genotypes (19 different strains), diversity at the nt level varied between 0·05 and 0·99% and at the aa level between 0·15 and 0·45%. When comparing only the two St1 strains, diversity at the nt level was 0·55% and at the aa level was 0·15%. Likewise, for St2 strains (17 different strains), the highest divergence was up to 0·55% at the nt and 0·3% at the aa level.

**Figure 4:**
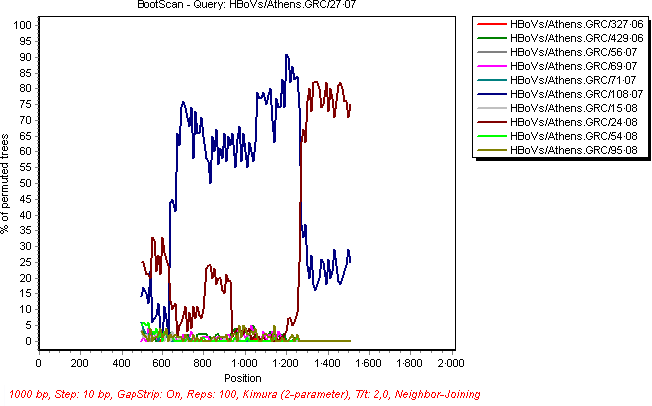
Applied bootscan in Simplot software to detect the recombination point between hBoV strains. The most likely breakpoint was located at position 1260 bp of the complete VP1/VP2 gene between a HBoVs/Athens.GRC/27.07-like and a HBoVs/Athens.GRC/24.08-like strain.

Interestingly, 5 aa substitutions were observed in both strains belonging to the third HBoV group and included L40S, A340P, G415S, T521N and F540Y while three additional mutations (P430L, L433F and P541L) were revealed in the strain HBoVs/Athens.GRC/108.07. The boot scanning analysis suggested that the strains HBoVs/Athens.GRC/35.07 and HBoVs/Athens.GRC/108.07 resulted from recombination between a HBoVs/Athens.GRC/27.07-like and a HBoVs/Athens.GRC/24.08-like strain (belonging to St1 group), with a breakpoint located at position 1260 bp of the complete VP1/VP2 region (Figure 4). Bidirectional sequencing, as well as, the fact that the recombination breakpoint was located within the 3rd amplicon of VP1/VP2 region suggested that this result was not due to mixed infection.

In conclusion, sequencing data suggested that at least 3 groups of HBoV cocirculated in Southern Greece during the study period. Similar observations were also made by Chieochansin *et al*.,^14^ Lin *et al*^19^ and Xu *et al*.^20^ In each of the above study populations, including ours, the representative strain of group 1 and group 2 was the prototype strains St1 and St2, respectively. The Greek strains, which clustered within group 1, were found to share a common aa substitution at position 590 (S590T, with respect to VP1 numbering) with all the St1 reference strains used in the phylogenetic analysis, compared with group 2 (Figure 3), an observation also made by others.^14^,^19^ Group 3 included 4 strains which came from Taiwan,^19^ Thailand^14^ and Greece. Additional substitutions at positions 415 and 540 (G415S and F540Y) found in the VP1 protein of Greek strains HBoVs/Athens.GRC/35.07 and HBoVs/Athens.GRC/108.07 had also been reported in two Italian strains.^21^

## Discussion

This is the first study on the genetic diversity of HMPV and HBoV strains in children with respiratory tract infections in Greece, including paediatric patients who suffered from ILI^11^ as well as other respiratory infections.

Since its initial documentation in the Netherlands in 2001, HMPV has been reported to circulate worldwide.^22^–^24^ We only found the B2 HMPV lineage circulating among the Greek population during the winter season of the three years examined, although cocirculation of all four viral lineages during the same period is not unusual.^25^,^26^ Reports from Croatia, Ireland and France have also indicated predominant circulation of lineage B2, during the winter periods of 2005–2006, 2006–2007 and the years 2006–2008, respectively.^27^–^29^ Gaunt *et al*^30^ suggested a potential back-shift to lineage B2 in Scotland during the winter season of 2007-2008. The observation of only one circulating lineage among the study population was also mentioned in other studies. In Spain,^31^ Italy^32^ and China^33^,^34^ circulation of HMPV A2 lineage was reported. However, a number of studies have indicated that circulating predominant strains may vary on an annual basis and that their replacement may occur, on average, every 1–3 years.^35^,^36^

As year-round surveillance was not performed and summer infections from HMPV and HBoV cannot be excluded, recirculation of the same HMPV lineage over three consecutive years within the Greek population could be the result of the maintenance of the virus throughout the year. Alternatively, HMPV lineage B2 could have been reintroduced each year in the Greek population with minor intralineage alterations causing infections to the naïve population.

Phylogenetic analysis of the HBoV full-length VP1 and VP2 proteins was performed on 26 HBoV-positive samples revealing limited sequence variations among them as well as in relation to available sequences of previously described HBoV strains. Genotype St2 was the only HBoV genotype, which circulated during the first winter period of 2005–2006, while both St1 and St2 cocirculated during the second and the third study period, with predominance of genotype St2. Interestingly, the majority of HBoV strains, detected in Italian children during winter periods of the years 2004–2008, also belonged to genotype St2 (62·5%).^21^ An even higher percentage (75·4%) of HBoV strains belonging to genotype St2 during the years 2002–2005 was reported from Germany.^37^

Our study exposed the circulation of a third group of HBoV strains during the second surveillance period. Lin *et al*^19^ and Xu *et al*^20^ also reported the circulation of HBoV strains belonging to a third group different from St1 and St2, which resulted from recombination events between different circulating viral strains. Such genetic recombination events have been reported in parvoviruses^38^,^39^ and in different HBoV species.^40^–^42^ Our data suggest that the strains HBoVs/Athens.GRC/35.07 and HBoVs/Athens.GRC/108.07 resulted from recombination events between a HBoVs/Athens.GRC/27.07-like and a HBoVs/Athens.GRC/24.08-like strains, with a breakpoint site in the VP1/VP2 region. To date, similar intraspecies recombination events involving HBoV1 have been reported before, with a breakpoint located in the NS1,^19^ VP1/VP2^21^ and NS1 and VP1/VP2^20^ regions. Taken together, these data suggest that intraspecies recombination could occur in HBoV1 at different positions of its genome. Further studies, including whole genome sequencing, are necessary to identify breakpoint patterns in these viruses, thus exploring their potential association with viral spread among populations.

Higher similarity observed between HBoV sequences to that observed between HMPV strains may be explained by the widespread assumption that mutation rates correspond to the fidelity of replication. DNA viruses, such as HBoV, mutate slower compared with RNA viruses, such as HMPV, because DNA polymerases possess proof-reading activity, which further reduces mutation rates during DNA replication.^43^ Moreover, the rate of generation and correction of genomic mutations may depend on a number of factors, including generation time, genomic architecture, replication speed, transmission and environmental effects.

Our study highlighted the heterogeneity of viral strains circulating within the Greek population by determination of the complete sequences of HMPV G gene and HBoV VP1/VP2 gene. The phylogenetic analysis performed aimed to monitor the genetic evolution of HMPV and HBoV during three consecutive years and revealed an individual predominant lineage for the former and a combination of genotypes for the latter.
